# The effects of dance interventions on physical function and quality of life among middle-aged and older adults: A systematic review

**DOI:** 10.1371/journal.pone.0301236

**Published:** 2024-04-19

**Authors:** Jingting Lu, Nur Athirah Abd Rahman, Matthew Wyon, Shazlin Shaharudin

**Affiliations:** 1 College of Physical Education, Hubei Normal University, Huangshi, China; 2 Exercise & Sports Science Programme, School of Health Sciences, Universiti Sains Malaysia, Kubang Kerian, Kelantan, Malaysia; 3 School of Sport, University of Wolverhampton, Walsall, United Kingdom; Novi Sad School of Business, SERBIA

## Abstract

**Background:**

Fundamental physical functions such as postural control and balance are vital in preserving everyday life, affecting an individual’s quality of life. Dance is a physical activity that offers health advantages across various life stages. Nevertheless, the effects of dance interventions on physical function, postural control, and quality of life among older adults have remained underexplored. The review aimed to examine the strength of evidence for dance interventions on physical function and quality of life among middle-aged and older adults.

**Methods:**

A systematic review was conducted across four databases (PubMed, Cochrane Library, Web of Science, and Medline), focusing on studies involving more than four weeks of dance interventions. MeSH terms [dance or dance intervention or dance rehabilitation or dance movement] and [motor function or functional capacity or postural control or functional mobility or mobility or postural balance or balance or flexibility or gait] and [well-being or quality of life or life satisfaction] were utilized in the search. This review was registered in the PROSPERO database (CRD42023422857). Included studies were assessed using the Cochrane Risk of Bias.

**Results:**

The search revealed 885 studies, and 16 met the inclusion criteria. The effects of various dance genres on physical functions and quality of life were compared. Most studies showed that dance intervention improved physical function, balance, postural control and quality of life. Dance intervention showed a high level of adherence compared to physiotherapy, self-care, conventional therapy, and aerobic and resistance exercise.

**Conclusion:**

In terms of improving physical function and quality of life, structured dance is a safe and relatively effective alternative to exercise. Note the effect of movement selection and intensity in the dance interventions. Dance with music may increase participants’ interest, encouraging more physical activity among middle-aged and older adults.

## Introduction

With the growing ageing population globally, the health and well-being of older adults have become increasingly important topics of concern [1]. This demographic shift has prompted a growing need to focus on the unique healthcare challenges and requirements faced by older adults [2]. As individuals age, potential health conditions become more prevalent. Aging is often accompanied by a decline in sensory, motor [3], and cognitive functions [4], which increases the vulnerability of older adults towards adverse health risks.

Age-related fundamental physical functions, such as postural control and balance [5], are crucial for preserving the well-being of middle-aged and older adults. Effective posture control ensures safe and stable movement, contributing to the overall physical function [6]. It has been observed that middle-aged and older adults often exhibit a reduced capability for postural adjustments required to regain stability [7]. Adequate posture control holds utmost significance in averting falls and injuries while also facilitating the execution of daily tasks, which contributes to enhanced quality of life (QoL) [8]. As a result, alternative and enjoyable avenues of physical activity to foster improved health results and elevate one’s holistic sense of well-being have gained growing attention.

Dance has received much attention as a potential intervention as it is a complex activity that combines physical exercise with cognitive, social, and artistic stimulation [9]. Dances are inherently multimodal, involving physical activity or exercise, learning, attention, memory, emotion, rhythmic motor coordination, balance, gait, visuospatial ability, acoustic stimulation, imagination, improvisation, and social interaction [10]. Available studies [10–13] explore how various types of dance affect individual health and cognition, but there have been limited research reviews on the effects of dance on physical function and QoL.

The current review aimed to evaluate the influence of dance interventions on physical function and QoL among middle-aged and older adults. The purpose was to discern potential variations in the effectiveness of dance interventions regarding the distinct health states of older adults and the different effects involving dance and other types of intervention (or no intervention). The questions of whether dance can emerge as a feasible and efficient intervention and its potential integration into diverse healthcare initiatives, rehabilitation centers, and community-based projects were considered.

## Methods

This review was registered in the PROSPERO database (CRD42023422857) and conducted according to the Preferred Reporting Items for Systematic Reviews and Meta-Analyses (PRISMA) guidelines [14].

### Search strategy

A systematic advanced search on PubMed, Cochrane Library, Web of Science, and Medline databases was conducted to identify full-text publications of eligible studies. The literature search was completed from inception until 1st May 2023. The search was limited to human studies and publications in English and Chinese based on the following medical subject headings (MeSH) search terms: [dance or dance intervention or dance rehabilitation or dance movement] and [motor function or functional capacity or postural control or functional mobility or mobility or postural balance or balance or flexibility or gait] and [well-being or quality of life or life satisfaction]. Besides that, reference lists of identified and included studies were manually searched for any studies not found in the database search. Inclusion and exclusion criteria for the review were determined a priori.

### Inclusion criteria

The inclusion criteria were randomized controlled trials and original studies with dance interventions with physical function and well-being outcomes such as posture control, functional mobility, balance, and quality of life (or life satisfaction); middle-aged and older adults (>40 years) participants with no restrictions on health status.; accessible full text in either Chinese or English; intervention duration had to be a minimum of four weeks. Dance in this study refers to the movement of the body in a rhythmic manner [12].

### Exclusion criteria

Conference proceedings or abstracts, editorials, commentaries, opinion-based papers review articles (systematic and narrative), case series, and case studies, studies with no control group, interventions that combined dance with other movement, and interventions that used dance primarily as a therapy for mental health issues were excluded.

### Data extraction

After the search, article titles and abstracts were initially screened against the inclusion/exclusion criteria based on population, intervention, control/comparator, and outcome (PICO, Table 1) by J.L. and N.A.A.R. The same authors independently assessed the quality of the included studies, whereby S.S. resolved any discrepancies between the reviewers. Then, J.L. extracted the following data from each included study: name of the author(s), year of publication, sample size, attrition (calculated as the proportion of dropouts from the initial sample size), participants’ characteristics, mean age, details on the intervention program, and outcomes measured.

**Table 1:** Population, intervention, control/comparator, and outcome (PICO).

Population	Middle-aged and older adults
Intervention	Dance
Comparator/control	Other types of intervention or no intervention
Outcome	Physical function postural control motor balance Well-being quality of life life satisfaction

### Risk of bias

J.L. and N.A.A.R. assessed the quality of the included studies using the Cochrane risk of bias tool (Review Manager 5.4) [15]. S.S. resolved any disagreements. The assessment was conducted based on the following domains: (1) random sequence generation; (2) allocation concealment; (3) blinding of participants and staff; (4) blinding of outcome assessment; (5) incomplete outcome data; (6) selective reporting; (7) other sources of bias. Accordingly, each included study was categorized as follows: (1) high risk; (2) low risk; (3) unclear (i.e., insufficient evidence) risk.

## Results

### Study selection

**Fig 1:**
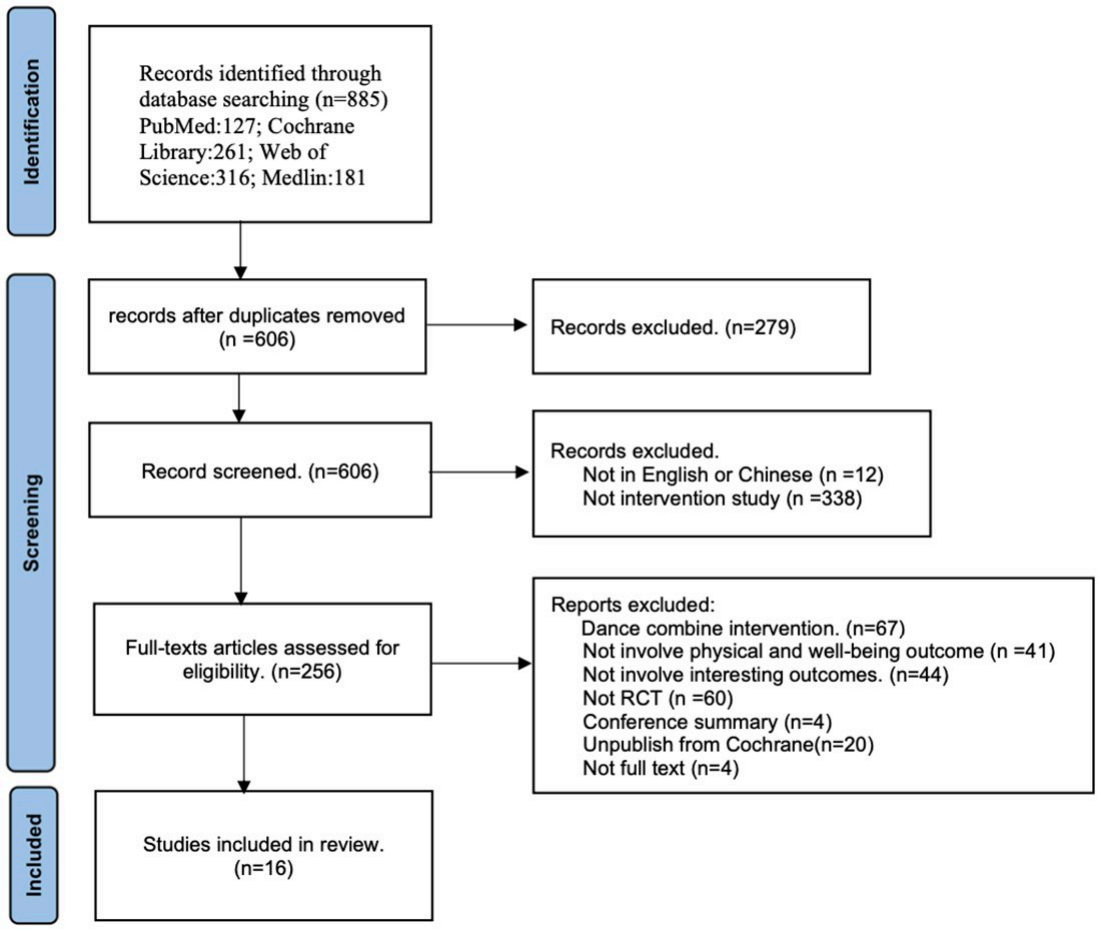
PRISMA flow chart of the study selection process.

The initial search resulted in 885 articles (Fig 1). After duplicates (279) were removed, there were 606 articles. Following the title and abstract review, 350 articles were further eliminated. Finally, 16 studies were deemed eligible to be included in this review.

### Research quality assessment

**Fig 2:**
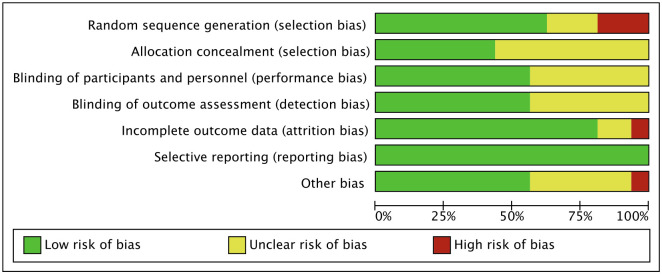
Risk of bias for randomized controlled trials included in this systematic review.

This systematic review included randomized controlled trials with varying degrees of quality as determined by the Cochrane risk of bias (Fig 2). The percentage of studies that reported low risk of bias for each parameter was 65% for random sequence generation [11, 16–23], 40% for allocation concealment [16–18, 20, 21, 23, 24], 50% for performance [11, 16, 18–24]and detection bias [11, 16, 18, 20–25], 85% attrition bias [11, 16–27]. Selective reporting was determined to have a risk of bias across all included randomized controlled trial studies (RCT). As for other sources of bias, there were both low risk of bias (60%) [11, 17–19, 21–23, 25] and unclear risk of bias (30%) [12, 16, 20, 24, 26–28]. Only one study [29] exhibited a high risk of bias due to the potential influence of a higher baseline activity rate in the control on the responsiveness to change.

### Characteristics of participants

The total sample size of all included studies was 1,259 participants, with males (212), females (968) and dropouts (79). In particular, the lowest sample size was 24 participants, and the highest sample size was 530 participants. Six studies [16–18, 25, 26, 28] included only female participants, whereas one study [19] included only male participants. Nine other studies [11, 12, 20–24, 27, 29] included male and female participants. In addition, 16 studies have different health conditions of the participants, including healthy elderly [12, 25, 27], Parkinson’s patients [11, 20, 23, 24, 29], patients with fibromyalgia [16], patients with chronic heart failure [19], patients with schizophrenia [21], postmenopausal women [18, 28], cancer survivor [26], and no record of health status [17, 22].

### Characteristics of interventions

Dance genres included Turkish folkloric dance [25], Waltz/Foxtrot [24], belly dance [16], Irish set dancing [20, 23], Agilando dance [27], Greek traditional dance [19, 21], creative dance program [17], Argentine Tango [24, 25, 29], social dance [22], Flamenco dance [18], ballroom dance [26], Indian folk-dance [12] and Zumba [28]. The shortest intervention duration was six weeks [12], and the longest was 12 months [22]. In addition, studies reported exercise frequency that ranged from one day per week [11, 20, 27] to five days per week [12, 26]. Meanwhile, the duration of class sessions ranged from 45 minutes [26] to 90 minutes [20, 23]. The class session duration was typically 50 minutes [17–19, 28] to 60 minutes [11, 12, 16, 21, 22, 24, 25, 27, 29].

There were 16 RCTs, with nine comparing a dance intervention group with a control group (no intervention) and seven comparing a dance intervention group with other activity groups (self-care, regular activity, or other physical exercises). In addition, one study involved a three-arm intervention [19], including an intervention group, an aerobic and resistance group, and a control group. One study compared four groups [24]: waltz/foxtrot group, Argentine tango group, tai chi group, and control group. The remaining fourteen studies involved dual arm interventions. The characteristics of the included studies are summarized in Table 2.

**Table 2:** Summary of characteristics included studies (N = 16).

Author(s)	Sample size	Gender	Participant’s characteristics	Mean age (SD)	type of dance	program duration, sessions per week	intervention				task	outcomes
(Eyigor et al., 2009) [25]	N = 40 group1 = 19 group2 = 18 dropout = 3	F	over the age of 65 healthy adult elderly no regular exercise training	group1 = 73.5±7.6years group2 = 71.2±5.5years	Turkish folk dance	8-weeks 3/per week 1h	group1: warm-up,10-min walk special folklore dance stepping, 40 min stretching and a cool-down,10 min		group2: did not have any exercise.		physical performance: (1) 20-m walk. (2) Six-min walk. (3) Stair climbing. (4) Chair rise The Short Form-36 questionnaire (SF-36) Berg Balance Scale (BBS)	Group 1: Statistically significant improvements were found in physical function (6-minute walk, chair, and stair climbing), BBS, and SF-36 subscales (physical function, general health, mental health) (p < 0.05) Group2: SF-36’s general health score has dropped significantly (p < 0.05)
(Baptista et al., 2012) [16]	N = 80 dance group = 40 control group = 40 dropout = 6	F	female patients with fibromyalgia	dance group = 49.5 years control group = 49.1 years	belly dance	16-weeks/32-weeks 2/per week 1h	dance group: warm-up predetermined dance movements choreography and cool-down exercise		control group: did not have any exercise		Functional capacity: the six-minute walk test Quality of life Fibromyalgia Impact Questionnaire (FIQ) the Quality-of-life Short Form 36 (SF-36)	The dance group shows Statistically significant differences on the six-minute walk test (p<0.001), FIQ score, and SF-36, the control group remained stable. The dance group achieved better results than the control group.
(Kattenstroth et al., 2013) [27]	N = 35 dance IG = 25 non-dancer CG = 10	IG(M/F = 8/17) CG(M/F = 3/10)	healthy elderly age range 60–94 years	dance IG = 68.60 ± 1.45 years non-dancer CG = 72.30 ± 1.84 years)	special dance program call Agilando	24-weeks 1/per week 1h	IG: warm-up: 20 min dance section: 40-min learn step sequences of increasing complexity.		CG: did not have any exercise		Posture and balance by a force platform	IG: Postural performance improved significantly among subjects in the IG group. No differences were found for subjects in the CG group.
(Volpe et al., 2013) [20]	N = 24 each group = 12	Irish dance (M/F = 7/5) Physiotherapy (M/F = 6/6)	• Mild to moderately idiopathic Parkinson’s disease (level 0–2.5 by medical)	PD Irish dance = 61.6 ± 4.5 years PD Physiotherapy = 65.0 ± 5.3 years	Irish set dancing	6 months 1/per week 1.5 hours	Irish dance: warn up (10 minutes), consisting of range of movement, balance, and postural exercises Irish dance lessons (70 minutes), reel and polka steps, and sets from different counties of Ireland. cool down (10 minutes)		Physiotherapy: warm-up range of movement and stretching exercises (10 minutes) strength training, balance training, and postural re-education (50 minutes) gait training (20 minutes) cooldown (10 minutes)		Motor disability by the Unified Parkinson’s Disease Rating Scale Motor Subscale (UPDRS) Time Up and Go (TUG) Berg Balance Scale (BBS) Freezing of Gait Questionnaire (FOG) The Parkinson Disease Questionnaire-39 items (PDQ-39)	Compare Analysis of variance between the two groups, UPDRS III motor section scores, the TUG test, and the FOG questionnaire shows better results for the Irish dancing group; the Berg Balance Scale and PDQ-39 showed similar outcomes.
(Cruz-Ferreira et al., 2015) [17]	N = 112 EG = 32 CG = 25 Drop out = 21	F	older females; age over 65 years; independent gait without an assistive device; nonexistence of cognitive impairment; absence of cardiovascular, neuromuscular, or neurological disorders	EG = 71.1 ± 3.9 years CG = 72.8 ± 4.5 years	Creative Dance program	3-months/6-months 3/per week 50min	EG = The creative dance sessions general mobilization (15 min), main phase (25 min), cool down (10 min).		CG = Maintain normal lifestyles during the duration of the study, including physical activity patterns.		Strength: the 30-s chair stand test. Aerobic endurance: The 6-min walk test. Flexibility: The chair sit-and-reach test. Motor agility/dynamic balance: The 8-ft up-and-go test. Life satisfaction: The Satisfaction with Life scale.	Physical Fitness: The EG had better physical fitness in strength, aerobic endurance, flexibility, and motor agility/dynamic balance compared with the CG. Life Satisfaction: At Weeks 12 and 24, the life satisfaction was significantly better for the EG than for the CG
(A. Kaltsatou et al., 2015) [21]	N = 51 Group A = 16 Group B = 15	Group A = 16 (M/F = 14/2) Group B = 15 (M/F = 11/4)	Sedentary patients with schizophrenia	Group A = 59.5 ± 19.6 years Group B = 60.4 ± 8.6 years	Greek traditional dance	8 months 3/per week 60 min	Greek traditional dancing program (Group A): warm-up (10 min), included stretching dance phase (40 min): consisted of basic, low impact steps, performed in a single group while holding hands in a hemicycle. cool-down (10 min)		Sedentary control group (Group B) had no formal intervention except from psychotherapy and continue their usual sedentary lifestyle.		Functional capacity assessments: (1) Six-minute walk test (2) Sit-to-stand test (3) Berg Balance Scale (4) Low limbs strength testing Mental assessments: (1) Quality of Life Enjoyment and Satisfaction Questionnaire	Group A showed significant increases in functional capacity In addition to left and right grip strength. Quality of life, and the Global Assessment of Functioning Scale score revealed an improvement, the Positive and Negative Syndrome Scale significantly decreased. Group B has no significant changes in all these functional capacity variables.
(Rios Romenets et al., 2015) [29]	N = 40 tango = 18 control = 15	Tango (M/F = 12/6) Control(M/F = 7/8)	Patients with idiopathic Parkinson’s disease.	Tango = 63.2±9.9 years Control = 64.3±8.1 years	Argentine tango intervention	12-weeks 2/per week 1h	Tango group: Each class consisted of a review of the previous class, plus a new step or elements, followed by improvisation activities.		The control group followed their usual schedule of pharmacological treatment. In addition, they were instructed to practice exercises that Exercise for people with Parkinson’s at home daily.		(1) Motor/Gait Movement Disorder Society Unified Parkinson Disease Rating Scale Mini-Balance Evaluation Systems Test (Mini-BESTest) Timed Up and Go and Dual-task Timed Up and Go Freezing of Gait Questionnaire (FOGQ) upper extremity function (2) Parkinson’s Disease Questionnaire-39	The MDS-UPRDS-3 was not significantly reduced in the tango group than in the controls. On motor outcomes Dynamic balance (Mini-BESTest) significantly improved in the tango group compared to controls (0.7 ± 2.2 vs. −2.7 ± 5.9, p = 0.032) and this difference remained significant even after multivariate adjustment for the baseline average time on exercise/dance (p = 0.013). There were no differences among groups on other nonmotor variables, including disease-related quality of life (PDQ-39).
(Merom et al., 2016) [22]	N = 530 dance group = 279 control group = 251 withdrew = 59 drop out = 47	Female: dance group = 231 control group = 217	older people resident of the village be able to walk at least 50 m	age>80 years dance group n = 119 control group n = 89	Social Dancing class	over 12-month 2/per week 1h (Total of 80 h, allowing for short breaks)	dance group: Folk dancing and ballroom dancing.		control group: continue regular activities		Functional mobility: the Short Physical Performance Battery (SPPB) gait speed the Physiological Performance Assessment (PPA) Health-related quality of life the self-reported SF-12 survey V2	study shows compared to the control group, folk dance participants performed significantly worse on the SPPB test and five chair rises; ballroom dancing seemed to improve their gait speed by 0.07 m/s, significantly more than control group whose mean gait speed declined (p = 0.05). Health-related quality of life has no significant different.
(Serrano-Guzmán et al., 2016) [18]	N = 70 Dance Therapy group (n = 27) Self-care Advice group (n = 25)	F	sedentary white postmenopausal women	Dance Therapy group = 69.07±4.41 years (60–78) Self-care Advice group = 69.48± 3.22 years (65–75)	Flamenco and sevillanas program	8-weeks 3/per week 50min	Dance Therapy group: Warm-up(10 min),mobility and low-intensity aerobic exercise Dance therapy (20 min), simple flamenco dance steps (forward, backward, transversal, and rotational), sevillanas, and ballet steps. Choreography(10 min),low-impact aerobics. Cooldown (10 min), Stretching relaxing.		Self-care Advice group: Follow physical activity recommendations.		the timed up-and-go the one-leg stance 12-item Short Form Health Survey (SF-12) Quality of life questionnaire	Balance scores were significantly better in the experimental group, and with moderate effects on physical activity and fitness; quality of life has not significance change. There was no significant change in the control group
(Pisu et al., 2017) [26]	n = 31 intervention = 15 control = 16 drop out = 2	F	at least 19 years old 3 months or more past their cancer treatment married or in a romantic relationship for 12 month or more not pregnant or planning to be pregnant	intervention = 56.7±8.6 control = 59±10	ballroom dance	12 weeks 5/per week 45 min	intervention group: Foxtrot, Waltz, Cha-Cha, and East Coast Swing		control group: received no intervention		Functional capacity the 6 Minute Walk Test Quality of Life the SF-36	Intervention group showed significant improvements in functional capacity (p = 0.03), in the mental component of quality of life (p = 0.01), as well as physical functioning marginally significant improvement. The Control group showed marginally significant improvement in functional capacity (p = 0.06). Two groups have no improvement in quality of life.
(Shanahan et al., 2017) [23]	n = 90 each group = 45 dance group = 20 control group = 21 drop out = 49	dance group(M/F = 13/7) control group(M/F = 13/8)	Individuals with idiopathic PD	dance group = 69±10 control group = 69± 8	dance class and home dance program	10 weeks 1/per week 1.5h 3/per week 20 min	The dance group: warm-up: targeting movement speed and size, postural alignment, and other physiological systems required for dance. dance part: the reel and hornpipe step. cold down: flexibility exercises		The control group:usual care and daily activities.		motor function the Unified Parkinson’s Disease Rating Scale Motor Subscale (UPDRS-3) functional endurance 39 (six-minute walk test) Mini-Balance Evaluation Systems Test (mini-BESTest). the quality of life The Parkinson Disease Questionnaire-39 items (PDQ-39)	There were no significant differences between groups comparisons in PDQ-39, six-minute walk test, and mini-BESTest. the DG showed minimal signs of implementing UPDRS-3, and the CG deteriorated. Intragroup comparisons improved non-significantly in both groups, yet the dance group improved to a greater extent. Endurance declined in both groups during the intervention but to a much larger degree in the control group.
(Rocha et al., 2018) [11]	N = 42 Argentine tango group n = 10 Mixed dance group n = 11 drop out = 3	Argentine tango group n = 10(M/F = 4/6) Mixed dance group n = 11(M/F = 4/7)	idiopathic Parkinson’s disease, rated I–IV. able to stand for at least 2 minutes. able to walk independently for more than 3 meters with or without assistive devices	Argentine tango = 70.2 (5.5) years Mixed dance = 72.9 (5.5) years	Argentine tango or mixed-genre dancing	8-weeks 1/per week 1h	Argentine tango group: warm-up(10min) dancing(45min) basic tango steps and simple dances cool-down(5min)		Mixed dance group: warm-up(10min) dancing(45min) Tap dancing, creative dance, and Irish dancing steps. cool-down(5min)		the modified Time Up and Go Test Berg Balance Scale Functional Gait Assessment Freezing of Gait questionnaire Movement Disorders Society Unified Parkinson’s Disease Rating Scale sections II and III 39-item Parkinson’s Disease Questionnaire	There were statistically significant differences between baseline and post-intervention scores for mobility, balance, and motor disability in the Argentine tango group. For the mixed-genre group, improvements in the freezing of gait between baseline and post-intervention were statistically significant. but there were no significant differences between the groups.
(Lahiani et al., 2023) [28]	N = 53 Zumba training group = 23 control group = 19 drop out = 4	F	Women with aged between 50 and 60 years with a maximum of 5 years being postmenopausal	Zumba group = 56.2 (3.8) years Control group = 55.9 (4.2)	Zumba dance	12-weeks 3/per week 50 min	Zumba group: warm-up: 5 minutes, slow and quick walking, easy Zumba elements to music, dynamic stretching; fast music to elevate heart rate from 50 to 60% of maximum HR. Zumba part: 40 minutes, forward, sideward, and backward steps, spinal rotations, combined with turns and little jumps. cool down with relaxation		Control groups were not subjected to any exercise program		Postural Balance: force platform Lower Limb Strength: the 30-s chair stand test Quality of Life: The Short Form -36 questionnaire	Postural balance: the CoP velocity values were significantly smaller (p < .05) in the ZG; no significant (p > .05) difference was found between the pre- and posttest sessions in the CG. Lower Limb Strength: ZG has significantly better performance; no significant difference was found in the CG. Quality of life: the Short Form -36 questionnaire- Quality of Life scores were significantly better in the ZG posttest; no significant difference was found in the CG.
(Mishra & Shukla, 2022) [12]	N = 40 Group A: Indian folk-dance = 20 Group B: Conventional therapy = 20	Group A:(M/F = 0/20) Group B:(M/F = 8/12)	healthy elderly; 60to70 years of age	Group A:64.9 ± 5.27 Group B:66.4 ± 5.37	Indian folk-dance therapy or Conventional therapy	6-weeks 5/per week 60min	Group A: warm-up: ROM exercise of all joints, 1 set of five repetitions each for 10 minutes. IFDT: low to moderate level of intensity and consisted of programmed choreography like ‘aathama’ (circle/spin of 8), ‘chattama’ (circle/spin of 6) and many more such movements that purely depends on rhythm and beats for 45 minutes. cool down: breathing and savasana exercise for 5 minutes.		Group B received conventional therapy exercise program according to the American College of Sports Medicine (ACSM) guidelines. 10 minutes of general joint mobility and range of motion exercises for all joints 2 sets of 10 repetitions. 30 minutes of brisk walking along with conventional balance training like weight shifts, one leg stance and tandem stance 10 minutes of breathing and relaxation exercise.		Fullerton Advanced Balance Scale Single leg stance test 6 Minute Walk Test The Short Form -36 questionnaire	There was a statistically significant improvement in both IFDT and the conventional group, but on the inter-group comparison, IFDT was better than the conventional group in balance, functional capacity, and quality of life.
(Kaltsatou et al., 2014) [19]	N = 57 Greek traditional dances (group A) = 18 formal exercise (group B) = 16 control group (group C) = 17 drop out = 5	M	• Greek male patients with documented heart failure II–III stage	group A = 67.2± (4.2) years group B = 67.1± (7.2) years group C = 67.2± (5.0) years	Greek traditional dances or aerobic and resistance exercise training	8-month 3/per week group A = 50 min group B = 60 min	Group A: warm-up (10 min): included stretching. dance phase (40 min): consisted of basic, low impact steps, performed in a single group while holding hands in a Semi-cycle. The intensity of the dances was low and increased.	Group B: stretching (10 min) aerobic exercise (20 min): stationary bicycle or a treadmill low and upper extremities resistance training (20 min): chest press, shoulder press, bicep curl, triceps extension and leg flexion and extension. Relaxation exercises (10 min)		Group C did not have any exercise	functional capacity tests Sit-to-stand test Berg Balance Scale Strength testing health-related quality of life The Greek version of the SF-36 Life Satisfaction Inventory	All the exercised patients at the end of the study showed significant improvements in their health-related quality of life results. The intrinsic Motivation Inventory was increased only in group A.
(Hackney & Earhart, 2009) [24]	n = 75 Waltz/Foxtrot = 17 Tango = 14 Tai Chi = 13 Not intervention = 17 drop out = 14	Waltz/Foxtrot (M/F = 11/6) Tango (M/F = 11/3) TaiChi (M/F = 11/2) Not intervention (M/F = 12/5)	Individuals with Hoehn and Yahr stages of I–III PD least 40 years of age could stand for at least 30 min walk independently three or more meters with or without an assistive device	Waltz/Foxtrot = 66.8±2.4 Tango = 68.2±1.4 Taichi = 64.9±2.3 Not intervention = 66.5±2.8	Waltz/Foxtro Tango Taichi	20 lessons within 13 weeks 2/per week 1h	Waltz/ Foxtrot: all dance steps in closed practice position.	Tango: all dance steps in closed practice position.		TaiChi: 37 postures of the Yang Short Style of Cheng Manching	Not intervention	the Unified Parkinson’s Disease Rating Scale Motor Subscale 3 (UPDRS-III) the Parkinson Disease Questionnaire-39 items (PDQ-39)

F: female; M: male; HRQoL: healthy related quality of life; IG: intervention group; CG: control group; PD: Parkinson’s disease; EG: experimental group; DG: dance group; ZG: Zumba group; CoP:center of pressure

### Measurements included in the studies

Table 3 presents measurements of physical function and QoL from the included studies.

**Table 3:** Summary of measurement tools in the included studies.

Physical Function Performance	Number of Study
20-m walk	1
Stair climbing	1
Chair rise	1
Six-min walk test	7
The time up-and-go test (TUG)	4
Sit to stand test	2
Strength test	2
The 30-s chair stand test.	2
The chair sit-and-reach test	1
The one-leg stance (OLS)	2
The Physiological Performance Assessment (PPA)	1
The Short Physical Performance Battery (SPPB)	1
Gait speed	1
The Unified Parkinson’s Disease Rating Scale Motor Subscale (UPDRS)	5
Freezing of Gait Questionnaire (FOG)	3
Functional gait assessment (FGA)	1
Upper extremity function	1
Balance	Number of Study
The 8-ft up-and-go test.	1
Force platform	2
Berg Balance Scale (BBS)	5
Fullerton Advanced Balance Scale (FAB)	1
Mini-Balance Evaluation Systems Test (Mini-BESTest)	2
Quality of Life	Number of Study
The Short Form -36 questionnaire	6
The Parkinson Disease Questionnaire-39 items (PDQ-39)	5
Fibromyalgia Impact Questionnaire (FIQ)	1
12-item Short Form Health Survey (SF-12) quality of life questionnaire	2
Life Satisfaction Inventory	1
The Satisfaction with Life scale.	1
Quality of Life Enjoyment and Satisfaction Questionnaire	1

### Physical function outcomes

In 16 studies, physical function outcomes were compared in nine studies [16, 17, 21–23, 25–28] between dance intervention and control group without physical activity (Table 4). Additionally, seven studies [11, 12, 18–20, 24, 29] compared physical function between the dance intervention group and the control group that incorporated physical activity (Table 5). 12 out of 16 studies highlighted slight to moderate improvements in physical function outcomes resulting from dance interventions. These enhancements were particularly noticeable in motor skills, such as increased gait speed and improved postural control encompassing balance, stability, gait, stride length, and sway. From the data extracted from these studies, it was observed that Turkish folk dance [25], belly dance [16], Agilando dance [27], creative dance [17], Greek traditional dance [21], ballroom dance [26] and Zumba [28] significantly improved physical function. In contrast, the physical function of the control group without physical activity showed no significant difference or decrease.

**Table 4:** Summary of physical function (the dance group versus the control group).

Author(s)	Outcome measure	Dance group(mean+SD)		Control group(mean+SD)		P-value
		pre	post	pre	post	
Eyigor et al., 2009 [25]	20-m walk	12.2 ± 1.6	11.9 ± 1.8	13.9 ± 2.3	14.6 ± 2.7	*Within the groups, p < 0.05. ≠between the groups, p < 0.05.
	6-min walk	419.1 ± 84.1	488.8 ± 51.2*,≠	402.2 ± 62.1	413.9 ± 69.4	
	Chair rise	10.3 ± 2.0	8.3 ± 1.0*,≠	10.8 ± 2.5	10.7 ± 2.5	
	Stair climbing	10.3 ± 1.8	9.2 ± 2.3*,≠	11.1 ± 2.7	10.9 ± 2.3	
	BBS	54.1 ± 2.2	55.3 ± 0.85*,≠	53.6 ± 2.1	53.9 ± 1.7	
Kattenstroth et al., 2013 [27]	Posture	0.41 ± 0.03	0.49 ± 0.04	0.55 ± 0.04	0.54 ± 0.04	IG:P = 0.001; CG:P = 0.247
A. Kaltsatou et al., 2015 [21]	Six-minute walk	227.1 ± 106.2	328.4 ± 35.9*	230.9 ± 53.4	238.0 ± 47.6	*p < 0.05, IG vs. CG
	Berg Balance Scale	45.4 ± 4.9	53.1 ± 2.1*	44.4 ± 6.7	43.2 ± 6.7	
	Sit-to-stand test	24.4 ± 2.1	19.1 ± 1.8*	24.8 ± 1.9	25.1 ± 1.4	
	Low limbs strength testing	44.5 ± 25.9	77.7 ± 25.7*	56.0 ± 31.7	51.0 ± 29.8	
Merom et al., 2016 [22]	PPA score	0.77±1.29	1.02±1.43	0.49±1.07	0.69±1.23	P = 0.31
	SPPB score	10.2 ±1.8	7.9±4.8	10.6±1.6	8.8±4.3	P = 0.21
	Repeated sit-to-stand	12.7±4.5	17.8±10.8	12.3±4.3	16.1±9.9	P = 0.19
	Gait speed	0.94±0.25	0.90±0.28	1.01±0.22	0.91±0.24	P = 0.68
Pisu et al., 2017 [26]	the 6 Minute Walk	466.7±73.4	517.4±68	454.5±95.2	474.4±76.7	P = 0.03*/ P = 0.06
Lahiani et al., 2023 [28]	Firm surface	EO:9.8±2.66 EC:11.44±2.75	EO:7.4±1.55* EC:8.23±1.5*	EO:8.08±2.53 EC:9.8±3.34	EO:7.6±1.48 EC:9.5±1.41	*P = .05.
	Foam surface	EO:17.9±5.4 EC:26.1±8.73	EO:13.3±2.31* EC:19.9±4.9*	EO:14.4±4.1 EC:22.7±6.3	EO:12.6±4.2 EC:25.7±6.5	
	the 30-s chair stand test	18.21±4.6	21.36±3.9*	17.42±4.5	16.81±5.1	p *< 0.05

Author(s)	Outcome measure	Dance group(mean+SD)			Control group(mean+SD)			P-value
		Basic line	16-week	32-week	Basic line	16-week	32-week	
Baptista et al., 2012 [16]	Six-minute walk test (m)	372.8±80.2	443.5±78.3	431±88.7	332±66.7	344.3±72.7	343±77.9	p<0.001
Author(s)	Outcome measure	Basic line	3-months	6-months	Basic line	3-months	6-months	
Cruz-Ferreira et al., 2015 [17]	30-s chair stand test	13.33±0.62	14.59±0.67	15.10±0.58	12.04±0.59	12.82±1.08	12.17±0.95	P = 0.093/P = 0.178/P = 0.010*
	The 6-min walk test	401.49±12.59	439.41±12.28	438.10±13.74	355.88±13.31	353.51±17.17	330.12±20.50	P = 0.051/P = .000*/P = .000*
	The chair sit-and-reach test	-3.15 ±1.70	1.47 ±1.17	3.17 ±1.36	-1.23 ±1.74	-3.43 ±2.40	-4.08 ±2.50	P = 0.198/P = 0.121/P = 0.029*
	The 8-ft up-and-go	7.98 ±0.44	7.19 ±0.39	6.57±0.20	8.41±0.53	8.42±0.48	9.01±0.76	P = 0.446/P = 0.006*/P = .000*
Author(s)	Outcome measure	Within dance group		Within control group		Between groups pre		Between groups post
Shanahan et al., 2017(only P value) [23]	UPDRS- III	P = 0.19		P = 0.12		P = 0.54		P = 0.07
	6-minute walk test	P = 0.56		P = 0.22		P = 0.57		P = 0.33
	Mini BESTest	P = 0.88		P = 0.57		P = 0.24		P = 0.28

BBS: Berg Balance Scale; PPA: physiological performance assessment; SPPB: short physical performance battery; UPDRS: the unified Parkinson’s disease rating scale; Mini-BESTest = Mini-Balance Evaluation Systems Test

**Table 5:** Summary of physical function (the dance group versus the other exercise group).

Author(s)	Outcome measure	Dance group(mean+SD)		Physical group(mean+SD)		P-value
		baseline	Post	baseline	Post	
Volpe et al., 2013 [20]	Motor UPDRS	24.58±3.87	17.42±3.85	23.93±3.50	21.00±3.07	DG:P<0.001/PQ:P = 0.001
	BBS	36.08±9.20	46.08±6.75	34.08±9.14	38.92±9.97	DG:P = 0.051
	FOG	11.42±2.78	4.92±2.07	10.75±3.39	10.16±4.47	DG:P = 0.000
	TUG	NR		NR		DG:P = .007
Rios Romenets et al., 2015 [29]	MDS-UPDRS	24.7±9.6	24.4±10.8	30.5±13.6	30.2±12.0	DG:P = 0.896/PG:P = 0.903
	Mini-BESTest	35.6±3.0	36.3±3.0	33.9±4.9	31.3±6.9	DG:P = 0.190/PG:P = 0.103
	TUG seconds	7.4±2.0	6.1±1.5	7.9±2.5	8.0±2.2	DG:P = 0.003/PG:P = 0.903
	Dual task TUG	1.1±0.6	1.5±0.7	1.5±0.6	1.3±0.7	DG:P = 0.042/PG:P = 0.082
	Dual task TUG, seconds	10.4±2.5	9.1±2.5	11.5±3.4	11.6±4.0	DG:P = 0.026/PG:P = 0.964
	FOG-Q	2.0±2.5	2.7±3.8	4.6±5.9	4.1±4.2	DG:P = 0.175/PG:P = 0.599
	Purdue pegboard(60s)	Left:19.6±3.5	Left:18.5±3.6	Left:17.7±4.3	Left:17.0±4.3	DG:P = 0.058/PG:P = 0.466
		Right:20.5±2.8	Right:19.7±2.7	Right:19.5±5.0	Right:18.9±4.7	DG:P = 0.120/PG:P = 0.607
		Both:14.4±2.0	Both:13.8±2.4	Both:13.1±2.9	Both:12.5±3.1	DG:P = 0.145/PG:P = 0.086
Serrano-Guzmán et al., 2016 [18]	TUG	10.08±2.41	8.29±1.39	10.36±2.20	10.44±2.09	DG:P = 0.022
	TUG manual	11.32±6.89	9.73±2.19	11.71±3.16	11.60±3.02	P = 0.189
	TUG cognitive	11.32±3.57	9.89±2.29	13.00±8.93	11.71±3.16	P = 0.02
	One-leg stance	7.14±3.80	14.7±5.95	7.20±3.02	7.24±3.20	DG:P = 0.001
Rocha et al., 2018 [11]	TUG	9.72±2.18	8.01±1.38*	9.82±3.21	9.57±3.12	*P<0.05: statistically significant difference
	FGA	23.5±5.73	24.75±3.95	22.50±7.74	23.20±6.69	
	FOG	9.49±6.70	6.88±6.68	7.80±6.42	5.30±4.87*	
	BBS	48.38±7.89	52.25±3.80*	45.30±10.40	50.00±5.90	
	UPDRS II	13.75±7.63	12.0±9.76	11.80±3.52	10.80±4.78	
	UPDRS IIIR	14.25±10.53	10.88±11.76	15.20±7.39	10.98±6.99	
	UPDRS IIIL	19.63±12.73	14.13±12.94*	17.30±6.70	12.70±6.61	
Mishra & Shukla, 2022 [12]	FAB	30±4.47	30.8±4.43*	31.4±3.55	31.65±3.48*	*P<0.05 different from per-post
	Single leg stance (EO)	13.79±6.28	13.86±6.15	14.23±5.84	13.96±6	
	Single leg stance (EC)	5.62±3.53	5.73±3.54	5.17±3.27	4.91±2.94	
	6 min walk distance	334.76±57.8	337.79±81.3*	397.55±16.81	409.2±17.31*	

Author(s)	Outcome measure	Dance group		Physical group		No intervention		
		pre	post	pre	post	pre	post	
A. C. H. Kaltsatou et al., 2014 [19]	Sit to stand test	18.0±1.8	14.3±1.1*(c)	18.8±2.0	14.7±1.2*(b)	19.2±3.8	19.3±3.7	*P<0.05 (pre vs. post) (b)P<0.05 (PG vs. NI) (c)P<0.05 (DG vs. NI)
	Berg Balance Scale	52.8±1.9	55.8±0.4*(c)	52.3±1.9	55.4±0.6*(b)	53.1±1.6	52.9±1.6	
	Strength testing	209.7±29.9	291.4±27.9	205.9±17.6	295.6±27.3*(b)	212.9±16.1	207.1±13.1	

Author(s)	Outcome measure	Waltz/Foxtrot		Tango		Tai Chi		Control		
		pre	post	pre	post	pre	post	pre	post	
Hackney & Earhart, 2009 [24]	Motor UPDRS III	26.9±2.5	NR	27.6±2.0	NR	26.3±2.5	NR	27.4±2.4	NR	P = 0.98

UPDRS: the unified Parkinson’s disease rating scale; BBS: Berg Balance Scale; FOG: freezing of gait; TUG: time up and go; MDS-UPDRS: movement disorder society unified Parkinson disease rating scale; FOG-Q: freezing of gait questionnaire; FGA: Fanfiction gait assessment; FAB: Fullerton Advanced Balance Scale; DG = dance group; PG = physical group; NI = no intervention; Mini-BESTest: Mini-Balance Evaluation Systems Test; EO: Eyes open; EC: Eyes close; NR = no report

Rocha et al. [11] compared Argentine tango against mixed dance genre intervention. Both interventions reported improvements in mobility, balance, and motor disability and the improved freezing of gait and QoL. Greek dance [19] and aerobic and resistance exercise exhibited the same benefits on physical performance and QoL, with no significant differences in the control group without intervention. An Indian dance intervention [12] reported more significant improvements in balance, risk of falls, physical function, and QoL than conventional therapy. Flamenco and Sevillanas [18] significantly contributed to better balance performance and physical activity levels than the other group that received self-care advice and physical activity. In a 2013 Irish dance study [20], Irish dance showed better outcomes in the Unified Parkinson’s Disease Rating Scale (UPDRS) motor section, Time Up and Go (TUG), and Freezing of Gait (FOG) compared to physiotherapy. These findings suggest that Irish dance improves movement disorders, gait, and balance.

It is worth noting that four studies showed opposite results. An analysis of the dance versus the control group [22] reported that the gait speed of the dance group increased (+0.03 m/s) while that of the control group decreased (−0.03 m/s), although the difference was not statistically significant. The results of the postural sway control group decreased by 9mm, but those of the dance group showed an increase of 3 mm postural sway. Folk-dance style showed worse score on the short physical performance battery test and five chair rises. In another study on dance and usual care for Parkinson’s disease [23], the dance group reported a minimal increase in UPDRS III scores, while the control group’s scores worsened. However, endurance decreased in both groups, with a more significant decline in the control group. One study [29] comparing home exercise and dance intervention found that motor and gait outcomes showed no significant improvement except for dynamic balance in the dance group. Another study [24] with a four-arm intervention (tango, foxtrot/waltz, taichi and no intervention) reported no significant differences in physical function outcomes.

### Quality of life outcomes

The results for quality of life (QoL) have been summarized in Tables 6 and 7. Ten studies revealed positive outcomes for QoL and life satisfaction following dance intervention. Among them, seven studies [16, 17, 21, 25–28] showed a significant improvement in the QoL for the dance group, while the control group’s results tended to remain stable or worsen. A study [17] focusing on life satisfaction demonstrated a significant improvement at 12 and 24 weeks, with no notable change in the control group. Two studies indicated a similar significant improvement in the QoL for dance groups compared to those undergoing aerobic and resistance exercises [19] and conventional therapy [12]. Regarding intergroup QoL, no significant differences were observed.

**Table 6:** Summary of the quality of life between the dance group and the control group.

Author(s)	Outcome measure		Dance group(mean+SD)		Control group(mean+SD)		P-value	
			pre	post	pre	post		
Eyigor et al., 2009 [25]	The SF-36 questionnaire	Physical functioning	79.1±18.9	88.8±12.2*, ≠	78.5±13.5	79.6±16.0	*Within the groups, ap < 0.05; ≠between the groups, p < 0.05.	
		Role—physical	66.2±38.5	76.5±38.0	80.8±34.1	69.2±44.7		
		Pain	62.4±27.3	72.7±19.7	60.3±24.0	54.1±20.3		
		General health	63.0±21.4	77.4±24.3*, ≠	72.0±21.0	64.5±21.1		
		Vitality	60.0±15.9	65.1±12.1	53.9±14.2	53.1±17.3		
		Social functioning	86.7±24.1	94.1±13.3	89.4±18.3	58.9±18.3		
		Role—emotional	56.8±36.8	72.5±39.5	58.9±30.9	64.1±28.8		
		Mental health	69.3±25.1	81.0±18.2*, ≠	73.9±15.6	71.7±16.1		
A. Kaltsatou et al., 2015 [21]	Quality of Life Enjoyment and Satisfaction Questionnaire	Total	29.8 ± 4.3	34.9 ± 5.2*	27.8 ± 5.1	28 ± 4.5	*p < 0.05, DG vs. CG.	
		Physical health	37.5 ± 2.9	41.8 ± 7.3*	34.9 ± 4.9	33.1 ± 4.0		
		Subjective feelings	37.3 ± 11.3	42.1 ± 10.6*	36.9 ± 5.3	38.9 ± 5.5		
		Leisure activities	15.0 ± 4.0	20.7 ± 5.3*	15.7 ± 3.3	16.9 ± 3.7		
		Household duties	15.2 ± 6.3	18.5 ± 7.1	14.0 ± 4.6	13.5 ± 5.6		
		Social relationships	24.5 ± 5.1	28.5 ± 4.8*	24.9 ± 6.2	25.3 ± 6.6		
		General activities	43.5 ± 6.5	46.1 ± 6.1*	40.2 ± 6.2	40.4 ± 6.3		
Merom et al., 2016 [22]	The self-reported SF-12 survey V2	Physical component score	43.0 ± 8.8	39.8 ± 10.9	44.3 ± 8.7	40.8 ± 10.8	P = 0.96	
		Mental component score	52.1 ± 8.4	49.4 ± 10.8	51.9 ± 7.6	50.3 ± 9.5	P = 0.34	
Pisu et al., 2017 [26]	SF-36	Physical Component	49.9 ± 9.7	52.0±4	46.7±10.3	44.6±9.9	DG:P = 0.67	CG:P = 0.17
		Mental Component	48.2±12.4	53.5±7.8	55.1±10.2	54.4±9.2	P = 0.01	P = 0.64
		General health	71.4±16.7	77.5±16.8	72.4±20.3	70.2±15.9	P = 0.21	P = 0.47
		Physical functioning	81.3±19	89.2±10.2	76.6±17.4	75.6±17.3	P = 0.09	P = 0.76
		Role—physical	66.7±39.8	92.3±21.4	75±34.2	65.1±36.3	P = 0.34	P = 0.29
		Bodily Pain	77.1±20.2	76.9±15.2	78.4±23.4	71.5±22.5	P = 0.23	P = 0.03
		Vitality	57.7±17.8	71±19.3	61.3±19.3	59.7±15.9	P = 0.004	P = 0.79
		Social functioning	82.5±23.5	93.3±12.1	89.8±17.8	85.2±20	P = 0.04	P = 0.41
		Role—emotional	66.7±39.8	89.7±28.5	91.7±25.8	87.5±26.9	P = 0.07	P = 0.16
		Mental health	76.0±16.6	78.9±12	82.5±15.8	82±13.1	P = 0.04	P = 0.8
Lahiani et al., 2023 [28]	SF-36	Physical functioning	82.9±8.3	88.2±3.9*	83.2±6.2	82.9±9.1	*: significantly different from pretest sessions at p < .05.	
		Social function	85.0±19.1	86.7±10.1	84.1±15.3	84.9±16.5		
		Mental health	66.5±17.9	84.9±10.4*	68.1±15.7	65.9±17.3		
		Pain	81.8±16.2	86.2±11.4	85.7±13.8	80.9±15.2		
		Health perception	42.6±14.6	88.8±30.1 *	45.1±6.9	44.8±7.5		
		Physical limitation	67.6±32.1	91.1±12.7*	65.4±21.9	62.9±23.6		
		Emotional limitation	72.9±36.3	89.7±21.0*	75.1±25.9	73.8±28.3		
		Energy/Vitality	68.2±21.9	74.1±13.2	66.3±17.5	69.5±20.5		
		total score	70.9±13.6	86.2±5.2*	71.6±13.5	70.7±13.2		
Kattenstroth et al., 2013 [27]	Subjective well-being in life		0.63 ± 0.05	0.65 ± 0.04	0.57 ± 0.08	0.58 ± 0.08	DG = 0.004 CG = 0.722	

Author(s)	Outcome measure		Dance group(mean+SD)			Control group(mean+SD)			P-value
			baseline	16-week	32-week	baseline	16-week	32-week	
Baptista et al., 2012 [16]	Fibromyalgia Impact Questionnaire		5.89±1.39	4.69±1.73	4.26±1.81	6.34±1.29	6.61±1.53	5.9±1.86	p<0.003†
	the Quality-of-life Short Form 36 (SF-36)	Functional capacity	44.9±1.89	52.9±21.1	56.3±19.9	32.6±18.9	33.1±18.6	39.1±22.0	p = 0.146
		Physical aspects	24.7±32.2	40.5±30.6	36.5 ±32.4	8.8±17.9	10.4±21.6	13.8±26.5	p = 0131
		Pain	29.6±17.5	44.7±20.7	46±19.2	25.7±13.4	25.1±14.2	29.1±21.1	p<0.001†
		General health	46±21.7	45±21.3	44.9±15.6	38±16.5	38.1±18.3	41.5±21.4	p = 0.580
		Vitality	41.3±18.8	50±22.8	47.6±23.8	29±18.2	30.7±18.1	37.1±21.8	p = 0.082
		Social aspects	52.6±27.7	64.1±28.0	57.2±27.0	47.6±23.1	47.6±24.5	51.3±25.5	p = 0.055
		Emotional aspects	34.2±36.90	55±33.6	51.9±39.6	21.2±33.1	17.5±26.1	31.5±38.7	p = 0.003†
		Mental health	46 ±19.9	54.2 ±20.7	52.3±20.8	43.4±24.0	44.5±26.6	46.2±22.6	p = 0.021†
Author(s)	Outcome measure		baseline	3-months	6-months	baseline	3-months	6-months	
Cruz-Ferreira et al., 2015 [17]	Satisfaction with Life scale.		24.03±1.36	26.59±1.23	27.94±1.15	23.50±1.42	22.54±1.53	22.71±1.44	Baseline: P = .765 3-months:P = .020 6-months:P = .002
Shanahan et al., 2017 (only P value) [23]	PDQ-39		DG:P = 0.88		CG:P = 0.18		Between group pre: P = 0.48		Between group post: P = 0.43

^†^ = significant P-values between groups; PDQ-39 = Parkinson Disease Questionnaire-39 items; DG = dance group; CG = control group; SF-36 = Short Form-36

**Table 7:** Summary of the quality of life the dance group versus the other exercise group.

Author(s)	Outcome measure		Waltz/Foxtrot		Tango		Tai Chi		Control		P-value
			pre	post	pre	post	pre	post	pre	post	
Hackney & Earhart, 2009 [24]	Parkinson Disease Questionnaire-39 items (PDQ-39)	Mobility^	29.12±2.17	24.27±2.17	29.82±2.39*	22.68±2.39	21.54±2.48	22.31±2.48	21.32±5.79	25.74±6.11	* = significant difference between pre and post within group, p < 0.05. ** Main effect of time, ^ Significant interaction between group and time.
		ADL	29.66±2.17	26.23±2.17	30.95±2.39	26.19±2.39	27.89±2.48	26.60±2.48	20.83±4.79	17.89±4.39	
		Emotional Well-Being	18.87±2.11	22.30±2.11	27.38±2.33	20.54±2.33	18.27±2.41	19.19±2.41	19.61±4.37	18.14±3.37	
		Stigma**	12.13±2.77	12.13±2.77	19.20±3.05	15.63±3.05	18.27±3.16	12.98±3.16	5.88±2.70	4.78±2.24	
		Social Support^	10.78±2.23	16.67±2.23	19.05±2.46*	11.91±2.46	14.10±2.55	8.33±2.55	6.37±2.32	6.37±2.63	
		Cognitive Impairment	30.88±2.29	29.78±2.29	35.71±2.52	29.91±2.52	32.21±2.61	36.06±2.61	27.57±4.55	22.06±4.12	
		Communication	27.94 = 2.17	30.88±2.17	23.81±2.39	20.238±2.39	24.36±2.48	30.13±2.48	18.63±5.13	15.69±4.51	
		Bodily Discomfort	27.94±3.24	25.49±3.24	30.36±3.57	29.17±3.57	28.22±3.71	37.82±3.71	32.84±6.29	30.39±5.98	
		PDQ 39 SI^	22.32±1.31	21.64±1.31	27.04±1.44*	20.03±1.44	23.11±1.49	24.66±1.49	19.13±3.26	17.63±3.06	

Author(s)	Outcome measure		Dance group		Exercise group		Control group		P-value
			pre	post	pre	post	pre	post	
Kaltsatou et al., 2014 [19]	The Greek version of the SF-36	physical health	40.3±2.3	43.2±1.1*b	40.3±2.3	43.2±1.1*b	40.7±1.9	40.1±1.6	* = P<0.05 baseline vs. follow-up b = P<0.05 group B versus group C c = P<0.05group A versus group C
		mental health	41.6±4.0	44.4±2.2*b	41.6±4.0	44.4±2.2*b	42.1±1.9	41.8±2.8	
		total score	81.9±5.2	87.6±2.7*b	81.9±5.2	87.6±2.7*b	82.7±4.2	81.9±3.7	
	Life Satisfaction Inventory		42.8±5.5	42.1±6.3	42.1±6.3	50.7±6.6 *b	42.7±3.1	43.2±3.6	

Author(s)	Outcome measure		Dance group		Exercise group		P-value
			pre	post	pre	post	
Volpe et al., 2013 [20]	Parkinson’s Disease Questionnaire-39		30.60±12.06	22.16±10.18	32.58±7.59	27.61±7.67	P = .153
Rios Romenets et al., 2015 [29]	Parkinson’s Disease Questionnaire-39		26.8±17.1	26.4±18.9	25.8±15.1	24.5±12.9	DG:P = 0.562; EG:P = 0.881 Interaction groups: P = 0.754
Serrano-Guzmán et al., 2016 [18]	12-item Short Form Health Survey	PCS	36.46±6.98	39.63±6.34	40.27±6.83	38.39±7.19	P = 0.064
		MCS	43.54±9.76	45.61±9.32	43.83±9.90	40.74±8.56	P = 0.064
Rocha et al., 2018 [11]	Parkinson’s Disease Questionnaire-39		22.53 ± 14.03	20.38 ± 12.60	27.37 ± 10.75	28.46 ± 7.75	DG:P = 0.46; EG:P = 0.664 Group effect: P = 0.677
Mishra & Shukla, 2022 [12]	SF-36	PCS	70.74 ± 13.13	80.07 ± 11.62*	73.47 ± 10.12	78.34 ± 9.17*	* Different from pre-test, p < 0.05.
		MCS	78.20 ± 9.98	84.30 ± 9.42*	82.12 ± 6.32	84.69 ± 6.42*	

PCS = Physical Component Score; MCS = Mental Component Score; SF = Short Form; DG = dance group; EG = exercise group

Six studies have reported different results. One study (dance vs. regular day life) [22] showed that both groups’ QoL scores decreased. Another study [23] showed a slight increase in QoL for the dance group but improved non-significantly in dance and the usual care group. Three studies [11, 18, 29] showed no significant differences between pre and post-intervention in both groups. A four-arm study (including tango, foxtrot/waltz, tai chi and no intervention) [24] demonstrated significant improvements in mobility (p = 0.03), social support (p = 0.05), and PDQ-39 Summary Index in health-related quality of life (HRQoL) for the Tango group, and no significant changes in HRQoL were noted in the waltz/foxtrot, tai chi or no intervention groups.

## Discussion

The current review focused on the effects of dance interventions on physical function and QoL among middle-aged and older adults. A total of 16 studies were identified; nine studies compared dance interventions and control groups without any intervention, while seven other studies compared dance interventions to different types of physical activity. The review indicates that dance has the potential to yield notably positive effects on physical function, particularly on postural control, balance, motor skills, and QoL. Regarding physical function, twelve studies reported the favorable exercise benefits of dance interventions for postural control, gait stability, and balance. Four studies found no significant differences before and after the sessions. Ten studies found substantial improvements in QoL following the dance interventions. Still, four other studies found no significant differences, and two other studies observed a decline in the quality-of-life outcomes.

### Effects of dance interventions on physical function and quality of life among middle-aged and older adults

Enhancing functional capabilities is paramount for middle-aged and older adults, particularly regarding their capacity to remain independent in daily activities. The included studies reported improved physical function and QoL among healthy middle-aged and older adult groups in Turkish folk dance [25], Agilando dance [27], and Indian folk dance [12]. Significant benefits were seen in motor skills (walking speed, gait), postural control and balance. Indian folk dance as an intervention significantly improved balance, fall risk, functional ability, and QoL, with similar or even better results than traditional therapeutic interventions [12, 30]. Indian dance repeatedly stimulates the somatosensory system and motor senses through dynamic changes in stretching postures, rotational movements, and basic footwork [30]. Unlike conventional therapy, this complex combination of movements can significantly enhance balance function [12]. Good balance is crucial for maintaining postural equilibrium, reducing the risk of falls and facilitating older adults’ day-to-day activities [31]. Balance impairments associated with mobility and daily activities can significantly contribute to a decline in the QoL for middle-aged and older adults [32], thereby diminishing their sense of well-being.

Studies included in the analysis explored two dance interventions for Parkinson’s disease: Irish dance [20, 23] and Argentine tango [11]. Dance interventions might have significant efficacy in assisting individuals with early- to mid-stage Parkinson’s disease. Volpe et al. [20] noted that dance intervention and physiotherapy positively improved physical function and QoL. The Irish dance group exhibited superior outcomes in freezing of gait, balance, and movement impairments compared to the physiotherapy group. Additionally, an interaction effect between intervention and time was observed in the dance group. No significant differences were found in the QoL between the two groups. In Shanahan et al. [23] study of Irish dance, the dance group showed improvements in motor function, QoL, and balance compared to the control group without and intervention. Despite the reduction in endurance observed in both groups, the decrease in the dance group was less than half of that in the control group, suggesting a protective effect in the dance group [23]. Better movement and balance were observed in the Argentine tango group, and gait freezing improved with the mixed-type dance. However, the Argentine tango and mixed dance groups had no significant differences in QoL [11]. Argentine tango and mixed dance steps are rhythm-oriented changes that move to the rhythm of the music, which provides alternative ways to adjust movement timing, improve physical performance and learn.

It is important to note the varying results of these studies. Hackney and Earhart [24] examined the QoL in patients with Parkinson’s disease who received waltz/foxtrot, Argentine tango, tai chi, and a control intervention. They reported significant improvements in overall health-related quality of life (HRQoL), specifically its relationship to mobility and support. At the same time, there were no significant changes in the Unified Parkinson’s Disease Rating Scale III in all groups. Compared to self-exercise at home, significant improvements in dynamic balance and gait for the Argentine Tango group were noted. Still, there were no significant differences in quality-of-life outcomes between both groups [29]. Argentine tango may not directly address movement in Parkinson’s disease symptoms, but it has shown promising results in improving balance. Argentine tango requires specific motor skills, including repeated start and stop motions, diverse movement speeds, rhythmic variation, and spontaneous multidirectional perturbations [33]. People with Parkinson’s disease may benefit from dancing classes that incorporate elements such as rhythmical music, large-amplitude fast movements, dancing with partners and step routines. It is crucial to encourage people with a chronic and progressive condition such as Parkinson’s disease to be physically active [34]. Participants consistently expressed that dance interventions were motivating and enjoyable exercises [23], further driving their interest in continued engagement. Nonetheless, a correlation has been identified between low-dose dance intervention and group-related advantages [11], with the effect size associated with the intervention duration. The acquisition of dance movements follows a progression from simple to complex and slow to fast, enabling participants to practice the movement sequence systematically. Prolonged intervention periods may promote automaticity in movement and intensify the overall impact [35]. Short-term intervention might not yield substantial improvements in physical function and QoL.

Also, we highlighted the benefits of dance intervention, including Flamenco Spanish dance [18] and Zumba dance [28], on postmenopausal women. These positive effects include mobility, balance, physical activity, and fitness improvements. The dance motions, such as jumping, spinning, and quick movement [36], induce neuromuscular adaptations [37], leading to increased strength of the lower limb and postural balance. The muscle strength gained from Zumba training may positively impact women’s physical function [38]. Indeed, among postmenopausal women, consistent engagement in exercise has been shown to contribute to the prevention of bone loss and improvement of balance and strength [39]. The study noted more pronounced improvements in the QoL of postmenopausal individuals in the Zumba dance group than those in the Flamenco Spanish dance group. Lelard et al. [40] reported a correlation between positive emotions and improved balance performance. Positive emotions may arise from the diverse range of Latin dance choreographies and energetic music used in Zumba class [41]. The previous study observed an equally improved QoL through Zumba training among older women [42].

The review noted favorable outcomes of dance interventions on fatigue and pain. It is known that pain, fatigue and physical activity are tightly intertwined [43]. 76% of fibromyalgia patients felt light activity worsened their pain and fatigue [42]. In the study, the dance group noted heightened pain and fatigue levels during the initial four-week period, which gradually diminished as time progressed and felt relaxed in daily life [16]. Fatigue significantly correlates with patients’ well-being, physical function, and QoL [44]. Based on the observations, fibromyalgia patients reduced pain from belly dance and improved performance in daily activities, which lasted for sixteen weeks post-intervention. Concurrently, significant enhancements were observed in the pain, emotional facets, and mental well-being dimensions of the Short Form-36, which had a positive effect lasting 32 weeks [16]. Fatigue is one of the most enduring long-term symptoms among cancer survivors that significantly disrupts their everyday functioning [45]. Among cancer survivors, ballroom dance has reduced fatigue and enhanced QoL, particularly physical activity and vitality. This results in improved relationships with partners and adaptability to return to normal life [26]. Dance can improve flexibility and muscle strength, thereby reducing feelings of pain and fatigue. A study with chronic heart failure also confirmed beneficial results in physical function with reduced fatigue when Greek traditional dance was included in a rehabilitation program [19].

Previous studies [46, 47] have consistently shown that patients with schizophrenia experience a reduced QoL, possibly due to the lasting effects of stigma and discrimination. When Greek traditional dance was added to rehabilitation programs for patients with schizophrenia, significant improvements in the functional capacity and QoL [21]. Since traditional Greek dance action in a sequence and coordinated to music can improve balance, the lack of improvement in walking ability may be due to insufficient intensity. Regardless of the health status of older adults, creative dance improved aerobic endurance, flexibility, and life satisfaction, with better outcomes for longer dance interventions [29]. However, a social dance program for seniors living independently in retirement villages did not exhibit better effects in terms of falls and QoL [26]. This result may be due to the chosen dance genres, social and folk dance, that did not incorporate the opportunity to stand unsupported or stand on one leg for an extended time [22].

As a form of exercise, dance increases health, maintains proper posture, stimulates the muscular system, and improves physical fitness [27]. The blend of rhythmic movement, self-expression, and cultural richness in dancing creates an inherently captivating and appealing experience that resonates with a broader range of individuals. This distinctive quality contributes to the heightened attractiveness of dancing compared to conventional sports activities. Dance positively affects physical function and postural balance, enhancing functional performance crucial for daily activities and improving QoL. Improved QoL through dance training has the potential to promote overall well-being and better health among middle-aged and older adults. After dance class, there is an improvement in subjective well-being and life satisfaction [48].

When engaging in dance therapy or exercise, middle-aged and older adults with varying health conditions may not necessarily prioritize the artistic elements of dance. Instead, dance aims to provide patients with physical and psychological benefits or offer healthy individuals diverse exercise options [45]. The participation of older adults in physical activities helps them maintain their physical health and allows them to interact with others. This interaction also removes the sense of loneliness, further stimulating their mental state and enhancing individual well-being [27]. Furthermore, dance interventions offer distinct advantages in patient compliance, yielding a lower attrition rate and facilitating long-term engagement. Dance can be inherently enjoyable and engaging, encouraging adherence to the program and minimizing the dropout rate. Additionally, dance’s dynamic and social aspects foster a sense of community and camaraderie, further motivating and sustaining engagement in dancing over an extended time. All these combined factors contribute to dance’s enduring appeal and effectiveness as a therapeutic intervention.

### Strengths and limitations

This comprehensive review delved into the nuanced impact of dance, as compared to the alternative forms of exercise and self-care, on both physical function and QoL among middle-aged and older adults. This systematic review included studies covering physical function (motor, postural control and balance) and QoL. Nonetheless, it is imperative to acknowledge certain limitations encountered in these included studies, especially the participants’ baseline health status and the consistency of implementing these interventions. These intricacies warrant careful consideration when interpreting the analyses and results in the current study’s systematic review. The integration of dance as an alternative means to traditional exercise programs holds the promise to improve the physical function of middle-aged older adults. This approach can improve overall fitness and elevate QoL among middle-aged older adults.

It should be noted that the quantification of therapeutic outcomes across distinct dance genres needs to be improved. Distinct dance genres inherently encompass diverse dance steps, each with unique movement patterns. For instance, actions like the body’s rotation and turn step in dance can stimulate the vestibular system, enhancing balance [12]. Similarly, engaging in single-leg standing dance steps can improve gait by increasing leg strength [25]. Different diseases and diagnoses also have potential implications for dance interventions.

Consequently, a meticulous approach is imperative when dance plans are personalized, considering the nuanced interplay between these dance movements and the specific manifestations of various diseases. A well-conceived dance regimen must aptly address the disease-specific concerns, ensuring efficacy and safety. Enhancing limb balance may involve a deliberate emphasis on lower extremity-focused movements, encompassing both unilateral and bilateral maneuvers. Such an approach facilitates neuromuscular flexibility within the lower extremities, promoting a comprehensive limb balance. This delicate balance between the specificity of movement and the physiological requirements is paramount in devising a dance plan that maximizes therapeutic outcomes. Additionally, the degree to which various musical elements contributed to the observed effects of these studies remains uncertain.

As for any systematic review, several limitations may arise, including heterogeneity among the study designs, varying dance styles, differences in outcome measures, and potential publication bias. Addressing these limitations, the review aimed to provide a comprehensive and impartial assessment of the current evidence on the effects of dance interventions on physical function and QoL.

### Recommendations

There are research areas found in the interpretation of this systematic review that warrant further investigation. First, there is a need to conduct a more in-depth exploration of participants’ regular exercise routines to prepare for dance sessions. Understanding the baseline physical activity levels can provide valuable context for interpreting the observed effects of dance interventions. Second, investigating participants’ hydration habits is essential, as this can significantly influence physical performance and overall well-being. Moreover, an enhanced examination of participants’ awareness of frailty, inability, and longevity could offer valuable insights into the holistic impact of dance interventions. Finally, future studies should include different groups with different diagnoses, as the potential impact of variables such as disease or diagnosis on the results obtained with dance interventions may need to be more accurate.

## Conclusions

This review has reported outcomes demonstrating that dance may improve physical function and QoL. These positive results reflect that dance is an effective and safe exercise alternative for middle-aged and older adults. Dance intervention may serve as a potential physical exercise for disease rehabilitation. Also, dancing with music may increase participants’ interest and encourage more physical activity among middle-aged and older adults. Further research and exploration are warranted to understand the diverse benefits of dance.

## Supporting information

S1 Checklist PRISMA checklist. (DOCX)
